# Genomic formation of Tibeto-Burman speaking populations in Guizhou, Southwest China

**DOI:** 10.1186/s12864-023-09767-7

**Published:** 2023-11-07

**Authors:** Jinwen Chen, Han Zhang, Meiqing Yang, Rui Wang, Hongling Zhang, Zheng Ren, Qiyan Wang, Yubo Liu, Jing Chen, Jingyan Ji, Jing Zhao, Guanglin He, Jianxin Guo, Kongyang Zhu, Xiaomin Yang, Hao Ma, Chuan-Chao Wang, Jiang Huang

**Affiliations:** 1 State Key Laboratory of Cellular Stress Biology, School of Life Sciences, Xiamen University, Xiamen, 361102 China; 2 Department of Forensic Medicine, Guizhou Medical University, Guiyang, China; 3 Shanghai Key Laboratory of Forensic Medicine, Shanghai Forensic Service Platform, Academy of Forensic Science, Ministry of Justice, Shanghai, 200063 China; 4 Department of Anthropology and Ethnology, Institute of Anthropology, School of Sociology and Anthropology, Xiamen University, Xiamen, 361005 China; 5 Department of Anthropology and Human Genetics, Ministry of Education Key Laboratory of Contemporary Anthropology, School of Life Sciences, Fudan University, Shanghai, China; 6 State Key Laboratory of Marine Environmental Science, Xiamen University, Xiamen, 361102 China; 7 Institute of Artificial Intelligence, Xiamen University, Xiamen, 361005 Fujian China

**Keywords:** Genetic structure, Admixture history, Gene flow, Tibeto-Burman, Guizhou, Southwest China, Tibetan-Yi Corridor, Southeast coastal

## Abstract

Sino-Tibetan is the most prominent language family in East Asia. Previous genetic studies mainly focused on the Tibetan and Han Chinese populations. However, due to the sparse sampling, the genetic structure and admixture history of Tibeto-Burman-speaking populations in the low-altitude region of Southwest China still need to be clarified. We collected DNA from 157 individuals from four Tibeto-Burman-speaking groups from the Guizhou province in Southwest China. We genotyped the samples at about 700,000 genome-wide single nucleotide polymorphisms. Our results indicate that the genetic variation of the four Tibeto-Burman-speaking groups in Guizhou is at the intermediate position in the modern Tibetan-Tai-Kadai/Austronesian genetic cline. This suggests that the formation of Tibetan-Burman groups involved a large-scale gene flow from lowland southern Chinese. The southern ancestry could be further modelled as deriving from Vietnam’s Late Neolithic-related inland Southeast Asia agricultural populations and Taiwan’s Iron Age-related coastal rice-farming populations. Compared to the Tibeto-Burman speakers in the Tibetan-Yi Corridor reported previously, the Tibeto-Burman groups in the Guizhou region received additional gene flow from the southeast coastal area of China. We show a difference between the genetic profiles of the Tibeto-Burman speakers of the Tibetan-Yi Corridor and the Guizhou province. Vast mountain ranges and rivers in Southwest China may have decelerated the westward expansion of the southeast coastal East Asians. Our results demonstrate the complex genetic profile in the Guizhou region in Southwest China and support the multiple waves of human migration in the southern area of East Asia.

**Supplementary Information:**

The online version contains supplementary material available at 10.1186/s12864-023-09767-7.

## Background

East Asia (EA) has great cultural, genetic, linguistic, and ethnic diversity. Many language families exist in EA, such as Sino-Tibetan, Austroasiatic, Hmong-Mien, Tai-Kadai, Austronesian, Indo-European, Turkic, Mongolic, Tungusic, Japonic, Koreanic, Yukaghiric, Chukotko-Kamchatkan [1–5]. The most prevailing language family in EA is Sino-Tibetan, with a huge population of approximately one-fifth of the world’s total human population size. Genetic studies in the past years have shed light on understanding the genetic history and language dispersal in EA [6]. Late Neolithic ancient DNA genomes from the Tibetan Plateau indicated the stable livelihood and genetic continuity in the past three thousand years on the Plateau [7–12]. The genetic legacy of Paleolithic hunter-gatherers was also found in the Y chromosome and mitochondrial DNA gene pool of the modern Tibeto-Burman populations on the Tibetan Plateau [13].

Modern Tibeto-Burman and Sinitic populations have common ancestral components from the Upper and Middle Yellow River Basin populations. The origin and expansion of Sino-Tibetan populations were suggested to be related to the development of the Yangshao and Majiayao cultures in the Neolithic Age with the rapid development of advanced millet agriculture. Studies of language genealogy have recently suggested that the Sino-Tibetan language family originated from the millet-farming groups in the Yellow River basin in Northern China [14, 15]. The divergence of the Sinitic and Tibeto-Burman groups took place approximately 6000 years BP in the Yangshao culture period [16]. The Majiayao culture in the Upper Yellow River was suggested to be associated with the ancestors of Proto-Tibeto-Burman populations. The Bronze Age Qijia, Kayue, Xindian, and Siwa cultures were also suggested to be related to the formation of the Tibeto-Burman-speaking populations in the northwest region of China [17–19]. Then some branches from the Upper Yellow River basin spread southwest toward the Tibetan Plateau, forming the present-day Tibetan groups. The archaeological investigation demonstrated that the permanent human occupation on the Tibetan Plateau happened at approximately 3600 years BP, after the development of sustained agriculture and mature domestication of animals [20, 21]. Moreover, the other branches from the Upper Yellow River migrated southward via the east margin of the Tibetan Plateau to the southwest region of China, forming the present Tibeto-Burman-speaking populations in the low-altitude region [22, 23]. These populations gradually expanded into the Indo-China Peninsula, South Asia, and Southeast Asia (SEA) [24–27].

Along the eastern edge of the Tibetan Plateau was the Tibetan-Yi Corridor (TYC), harboring several ethnically and linguistically diverse human populations. This region has numerous rivers and colossal mountain ranges, mostly from north to south and with descending altitude. It was an important region for West China's north-to-south interactions of various human populations. It covers 0.88 million km^2^ and three provinces of China, including Gansu, Sichuan, and Yunnan (from north to south). The most famous ethnicities within this region were the Han, Tibetan, and Yi; hence, it gained the renowned name “Tibetan-Yi Corridor”. The TYC was believed to play a major role in the dispersal of Tibeto-Burman-speaking populations. Many studies on whole genomes from the TYC region have been focused on articulating the contribution of Tibetan and Han ancestries. Yao et al. analyzed genome-wide SNPs from 10 Tibetans and 10 Han Chinese from the northern Tibetan-Yi Corridor region, concluding that TYC populations are a mixture of ancestries related to Tibetans on the Tibetan Plateau and surrounding EA groups in the low-altitude area [27]. A recent study suggested that an ancient population unrelated to the migration of the Tibeto-Burman language but perhaps related to an ancient southern EA ancestry had played a major role in the peopling of the region of south TYC [28]. Populations in mainland SEA, such as populations in Vietnam and Cambodia, are closer to these southern TYC populations. According to the literature, the southward expansion of the Nanzhao Kingdom was thought to have brought Tibeto-Burman speakers into SEA [29]. It was an important event to promote the southward expansion of the Tibeto-Burman-speaking populations.

Since the Ming Dynasty, the TYC began to be a bridge between the Tibetan Plateau and the more eastern populations of China. During the Qing Dynasty, from the 18^th^ to 20^th^ centuries, more Han people migrated to this region due to the developing interactions and increased defences on the western border of the Sinitic kingdom. Therefore, populations in this region also have a strong genetic affinity with Han Chinese populations. Of the 56 officially recognized ethnic human groups of China, 20 could be found in this region, including some Tibeto-Burman speakers. Bai, Qiang, and Yi groups are typical of them. Bai, Qiang, Yi, and Han groups were on the same branch of the clustering tree [23]. Bai group has a rich history local to the TYC region. The Chinese literature described the Qiang group as early as the Shang Dynasty. Moreover, the Yi group is one of the largest ethnic groups in the Tibetan-Yi Corridor, with an ancient history tied to this zone [30]. These three groups were the typical Tibeto-Burman-speaking populations in the TYC region. The Bai, Qiang, and Yi groups showed high connections to the Han group in the south. The Hani, Lahu, and Jino in the southern region of TYC are predominantly of southern EA ancestries. Some studies suggested that southern EA ancestries have also contributed to the population genetic structure in the TYC region. Recent genetic analysis portrayed a gradual change in genetic relationships from north to south in West China. In the southern TYC region, the populations were influenced by gene flow from mainland SEA and the southeast coastal region of China.

Yunnan was in the southern TYC region. A multi-layered process with population migrations and linguistic and cultural interactions reshaped the genetic structure and ethnolinguistic landscape at the crossroads of EA and SEA since the Neolithic. A recent study in Yunnan suggested that the genomic legacy of populations associated with Neolithic millet farming was primarily preserved in North Yunnan, especially in the Tibeto-Burman-speaking populations [31]. The high frequencies of haplogroup O2a2b1-M134 and its sub-clades in the Tibeto-Burman speakers in Yunnan supported millet-farming, driving Neolithic migrations from the Upper Yellow River basin to the south [19]. The genetic profile of Yunnan Tibeto-Burman speakers was consistent with archaeological and ethnolinguistic evidence, which showed the early population migration and cultural interaction from Majiayao Culture and later Qijia Culture from the Upper Yellow River basin to Yunnan. Similarly, the Xinguang Culture in Dali showed a strong connection with the Neolithic culture from the northwest region of China, supporting the hypothesis of the North China origin of the Sino-Tibetan populations. They suggested that the southward migration of Yellow River farmers followed the TYC in the eastern edge of the Tibetan Plateau principally.

In the southern TYC, the genetic structure and cultural landscape of the southern EA and SEA had been deeply influenced by rice-farming expansion from east to west since the Middle Neolithic. The influence of the rice-farming groups’ expansion might have followed both inland and coastal routes. The current Tibeto-Burman-speaking populations in Yunnan were mixes of three genetic components, including Yellow River millet-farming groups in the north, mainland SEA rice-farming groups, and the Southeast coastal groups. Therefore, multiple large-scale migrations from Northern China, southeast EA, and mainland SEA have significantly reshaped the genetic profile of the southwest region of China.

Guizhou province was in the northeast of the Yunnan-Guizhou Plateau, and it was in the east of TYC. Many huge mountain ranges and rivers existed between Guizhou and the TYC region. In the southward expansion of the Tibeto-Burman populations, some branches migrated eastward to the Guizhou region. Many Tibeto-Burman-speaking populations live in Guizhou [32, 33]. This interactional zone was associated with the expansion of the millet farming groups from the Yellow River basin and rice farming groups from the Middle and Lower Yangtze River plain [34, 35]. Multiple north-to-south and east-to-west human migrations occurred from the Early Neolithic to the Iron Age [36, 37]. Previous research mainly focused on Tibetan populations in the high-altitude region and the Tibeto-Burman-speaking populations in the TYC region. However, the investigations for the Tibeto-Burman-speaking populations in Guizhou (TBG) are scarce. Our understanding of them is limited because of the need for more sampling of the TBG [38–42].

The lack of dense sampling and high-density genome-wide data limited our understanding of the genetic profile of the TBG. Therefore, we collected DNA from 157 saliva samples from (TBG), including Bai, Qiang, Yi, and Tujia groups. Then, we genotyped these samples with 700,000 genome-wide single nucleotide polymorphisms (SNPs) arrays. We explored the genetic structure and uncovered the admixture history of the TBG groups.

## Materials and methods

### Ethics statement

Our study and sample collection were reviewed and approved by the Medical Ethics Committee of Guizhou Medical University and Xiamen University and were in accordance with the recommendations provided by the revised Helsinki Declaration of 2000. The participants provided their written informed consent to participate in this study.

### Sample collection

**Fig. 1:**
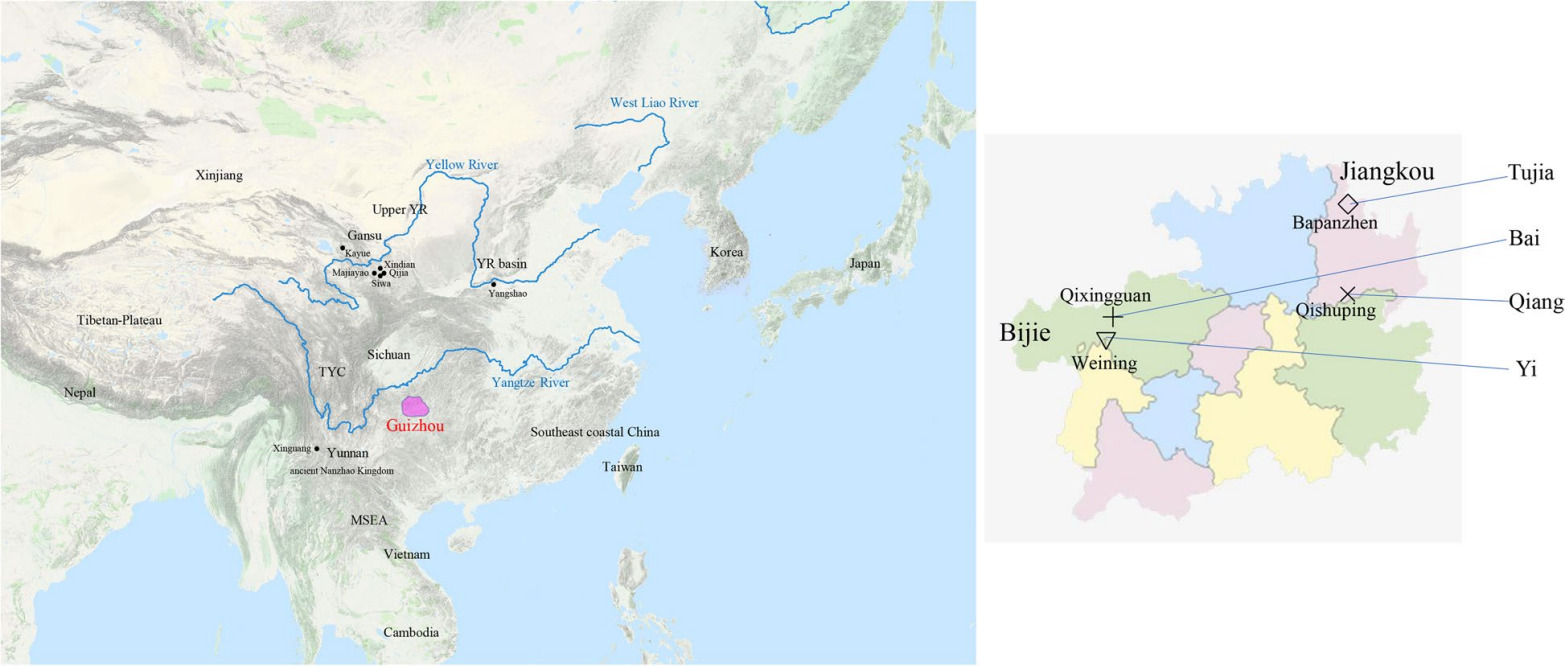
Geographic locations of TBG groups and other related regions in EA and SEA

We collected a total of 157 saliva samples from the TBG, including 50 samples from the Bai ethnic group in Qixingguan in Bijie, 11 samples from the Qiang ethnic group in Qishuping in Jiangkou, 45 samples from the Yi ethnic group in Weining in Bijie, as well as 51 samples from Tujia ethnic group in Bapanzhen in Jiangkou. The ethnicities of all participants were used as their self-declaration based on their family migration history and corresponding family records. The sample collection region is presented in Fig. 1.

### Genotyping and data preparation

We used PureLink Genomic DNA Mini Kit (Thermo Fisher Scientific) to extract DNA and measure the concentration via the Nanodrop-2000, following the manufacturer’s instructions. All these qualified samples were genotyped using the Illumina WeGene Arrays, covering about 700,000 SNPs at the WeGene genotyping centre in Shenzhen. We used Plink v1.9 [43] software to perform the quality control. We carried out the quality control by some parameters: –check-sex; –geno 0.05 –hwe 1e^−6^ –maf 0.01 –mind 0.05. Then, we removed the samples with inbreeding coefficients F > 0.0625. We removed individuals whose third-degree or closer relatives with PI_HAT > 0.125. After the quality control process, 440,036 SNPs and 127 TBG individuals remained. Of the remaining 127 TBG individuals, 44 samples were from the Bai group in Qixingguan in Bijie, 9 samples were from the Qiang group in Qishuping in Jiangkou, 27 samples were from the Yi group in Weining in Bijie, 47 samples were from the Tujia group in Bapanzhen in Jiangkou. Then, we merged our TBG samples with the previously published data of the ancient and modern populations mainly in EA and SEA [6, 22, 28, 31, 36, 37, 44–46]. After merging with the Human Origin dataset, 65,178 SNPs remained. We then merged our TBG data with the ancient individuals in the Guangxi region [46] and recently published old individuals on the Tibetan Plateau [12], and then 63,320 SNPs remained. In the end, there were 127 studied TBG samples and 1,217 reference samples. Detailed information on the reference populations has been listed in Supplementary Table 10.

### Principal component analysis

Principal Component Analysis (PCA) was performed using the *smartpca* package v16000 [47]. The PCA analysis (63,320 SNPs) was carried out at the individual level to describe the genetic structure of our TBG samples and the reference individuals. The numoutlieriter: 0 and lsqproject: YES options were applied for projecting ancient individuals onto the first two components calculated by modern individuals. We visualized the PCA results using the ggplot2 package v 3.4.1 in the R software v 4.2.2 (http://www.r-project.org).

### ADMIXTURE

For pruning SNPs with strong linkage disequilibrium in Plink [43], we carried out the analysis with the parameters “-indep-pairwise 200 25 0.4”. Then we ran ADMIXTURE v 1.3.0 [48] (After pruning: 53,350 SNPs) with the tenfold cross-validation (-CV = 10), varying the number of ancestral populations between K = 2 and K = 20 in 100 bootstraps with different random seeds [48]. We chose the best run according to the highest log-likelihood with the lowest CV error.

### *f*-statistics

We computed *f* statistics using ADMIXTOOLS with the default parameters and calculated standard errors (statistical significance) using a block jackknife resampling across the genome. We carried out *f*_*3*_*-*statistics by the *qp3Pop* v 651 and *f*_*4*_-statistics by the *qpDstat* v 980 programs [44]. We computed outgroup *f*_*3*_-statistics (63,320 SNPs) of the form *f*_*3*_ (X, Y; Mbuti) to measure the shared genetic drift between population X and Y since their separation from an outgroup Mbuti from Africa. We conducted the heatmap visualization of outgroup *f*_*3*_ values using the pheatmap package v 1.0.12 in the R software. In the *f*_*4*_-statistics (65,178 SNPs), a significantly non-zero value of *f4* (A, B; C, D) suggests that populations A and B (or C and D) are not consistent with being descended from a homogeneous ancestral population that split may earlier from the ancestral populations of the other two populations. A significantly positive value of an *f*_*4*_-statistics of the form *f*_*4*_ (A, B; C, D) indicates an excess allele sharing between population A and C or population B and D, but a significantly negative value of it suggests an excess allele sharing between population B and C, or population A and D. The Test in our *f*_*4*_ statistics mean the TBG groups.

### Genetic homogeneity testing by pairwise *qpWave*

We used pairwise *qpWave* v 810 as implemented in ADMIXTOOLS [44] to test whether pairwise populations were genetically homogeneous in relation to a set of outgroups (65,178 SNPs). A *p*-value > 0.05 for rank = 0 suggests that the two populations are genetically homogeneous relative to a set of outgroups. A *p*-value < 0.05 for rank = 0 indicates that a minimum of two streams of ancestry were needed to relate pairwise groups to the outgroups. We presented *p*-values of the results at rank = 0 with a heatmap by R software.

### Streams of ancestry and the inference of admixture proportions

We investigated the number of streams of ancestry, plausible admixture sources, and corresponding proportion using the *qpAdm* v 810 in ADMIXTOOLS [44]. We used the *f4-*based admixture modelling to investigate whether a batch of target populations was consistent with being related via N streams of source populations from a basic set of some outgroups. We calculated the admixture proportions of the given source populations quantitatively.

### Fst calculation

The Fst values were calculated by the *smartpca* [47]. We ran the *smartpca* with the parameters inbreed: YES and fstonly: YES, then output the results by phylipoutname parameter (63,320 SNPs). Then, we plotted a phylogenetic Neighbor-Joining (NJ) tree (NJ tree) using the Fst values of the populations in EA by applying the NJ algorithm in the MEGA software v7 [49].

## Results

### Population genetic structure in Guizhou province in Southwest China

**Fig. 2:**
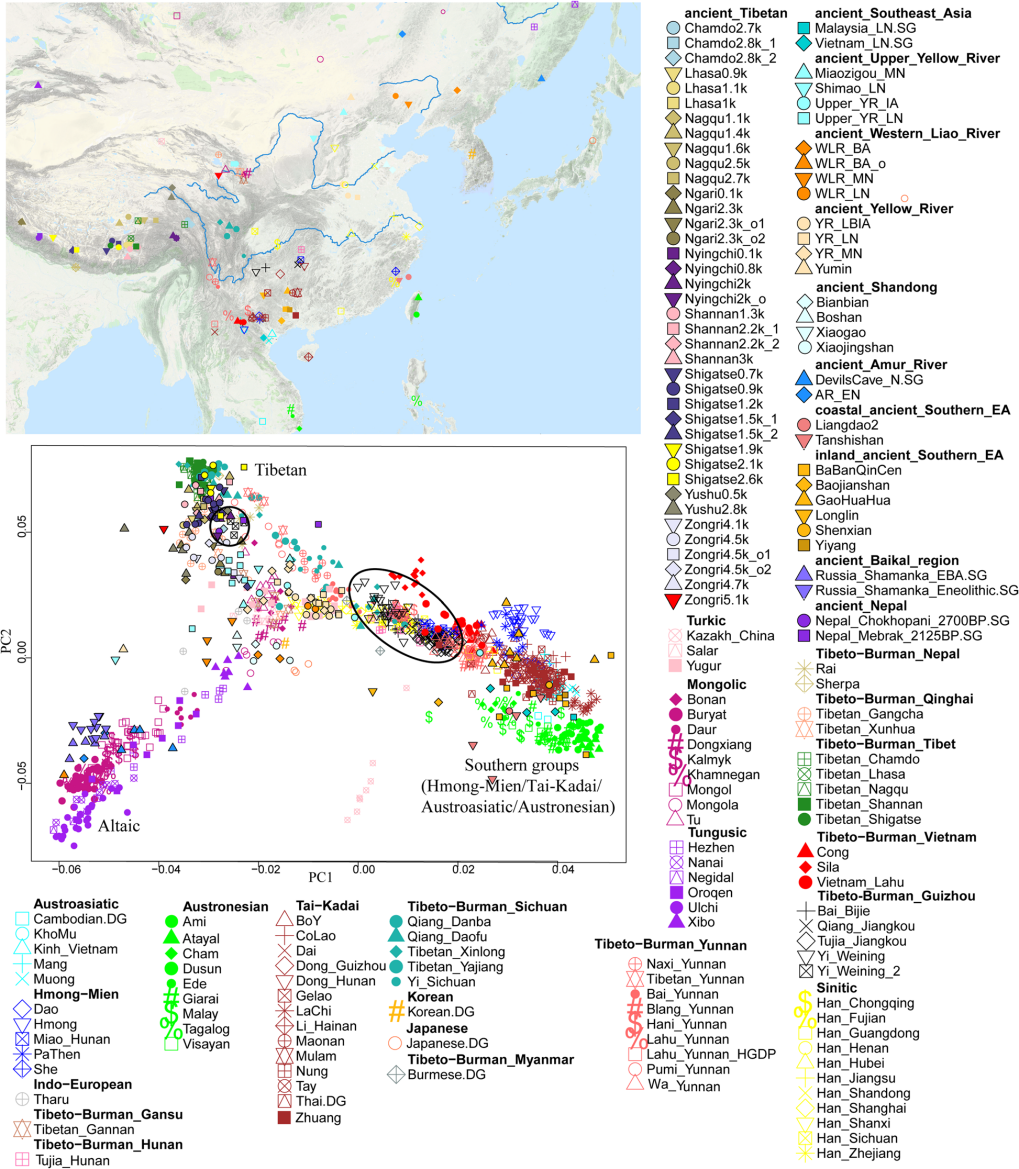
PCA plot of TBG with modern and ancient reference populations in EA and SEA. TBG groups are encircled. The map indicates the geographic locations of the populations included in the PCA analysis

We first carried out a PCA to explore the genetic substructure of EA and TBG (Fig. 2). From the PCA pattern, we found several genetic clusters correlating very well with the geographic and linguistic categories in EA, including one Altaic-speaking cluster consisting of Turkic, Mongolic, and Tungusic-related groups in north China; a southern cline with Tai-Kadai, Hmong-Mien, Austroasiatic, and Austronesian speaking populations; a genetic cline of Tibeto-Burman speaking populations, especially a Tibetan cluster. The Han Chinese cluster was in the center of the pattern. Our newly genotyped TBG samples were at an intermediate position in the cline of high-altitude Tibeto-Burman and low-altitude southern populations, such as the Austroasiatic and Austronesian groups, showing evidence of possible admixtures. However, we found four outlier individuals from the Yi group clustering with the high-altitude Tibetan people. We relabeled them “Yi_Weining_2” to distinguish them from the other Yi individuals shifting toward low-altitude southern populations.

**Fig. 3:**
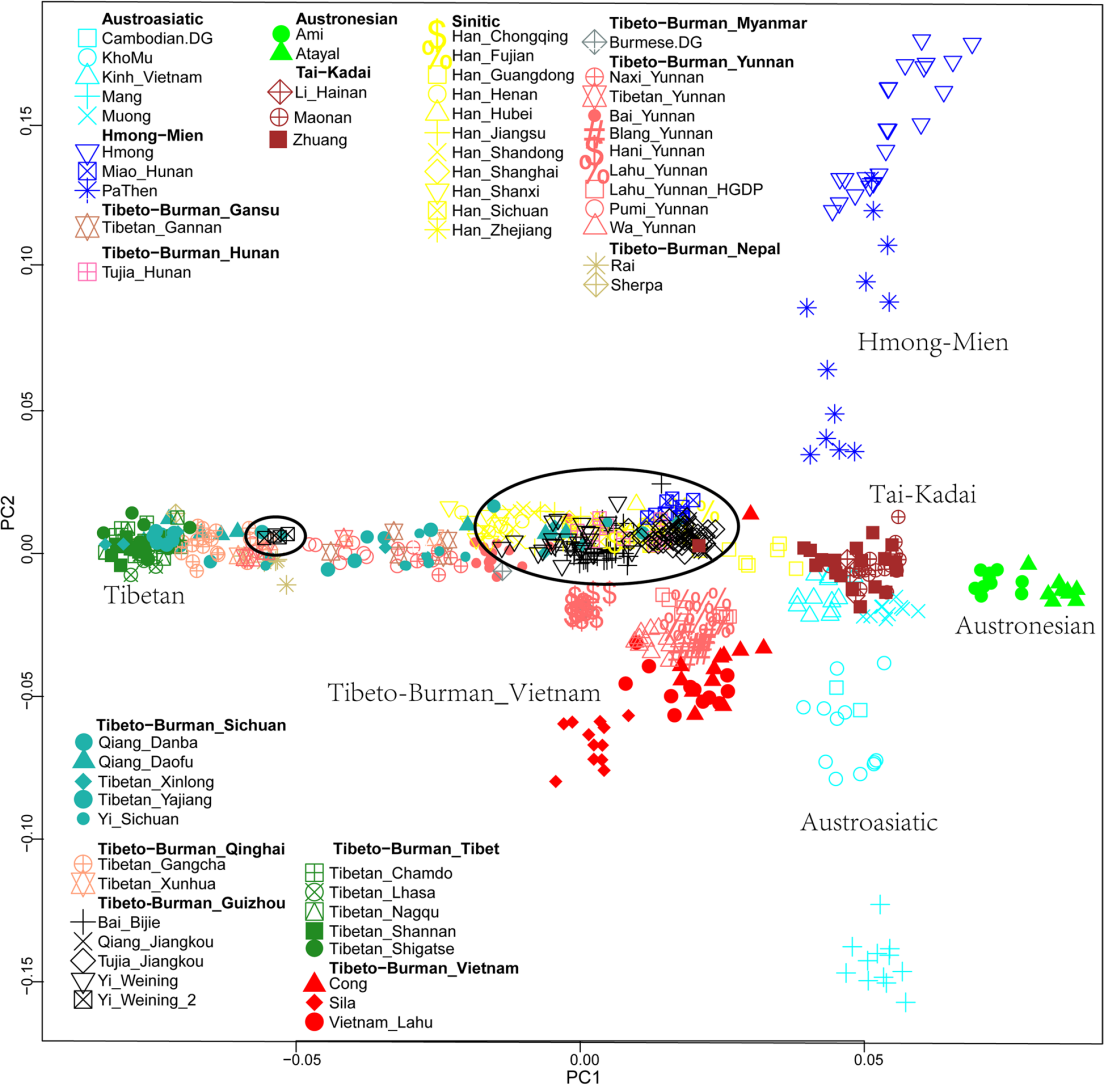
PCA plot of TBG and reference groups without northern and ancient groups. TBG groups are encircled

In the PCA pattern in Fig. 2, many individuals were very compact to each other, resulting in an indistinct view. Next, we removed the ancient and northern groups in EA from the PCA. Then, we carried out a more distinct PCA pattern (Fig. 3). Our newly reported TBG were between the Han Chinese and the southern groups in EA, including Austroasiatic, Austronesian, Tai-Kadai, and Hmong-Mien-related populations. It showed that the TBG might be the admixtures between Han-related groups and southern groups.

**Fig. 4:**
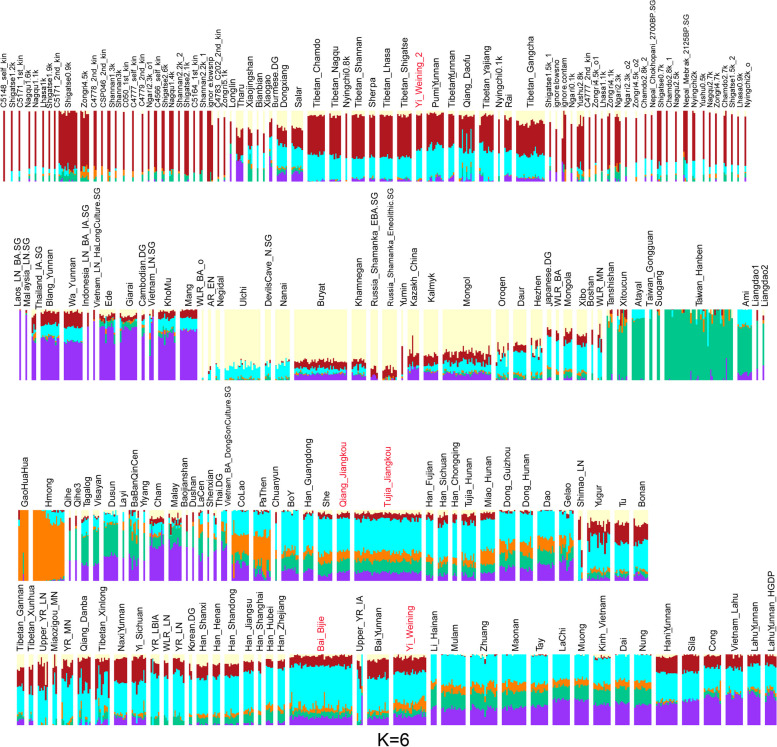
ADMIXTURE analysis result at K = 6. TBG groups are in red text

We next performed the model-based ADMIXTURE clustering analysis. We observed the lowest CV error at K = 6 (Fig. 4). At K = 6, we observed a red component primarily enriched in the Tibetan group, a purple component mainly found in Austroasiatic or proto-Austroasiatic populations, a faint yellow component distributed primarily in the Altaic populations in the north, a green component predominantly existed in Austronesian-related human groups in the southeast coastal region of China, an orange component enriched in the Hmong-Mien populations, and a blue component primarily distributed in the Han Chinses. Our TBG predominantly had the Han Chinese-related ancestry component. The TBG are genetically like other Tibeto-Burman-speaking populations and Han Chinese in Southern China.

**Fig. 5:**
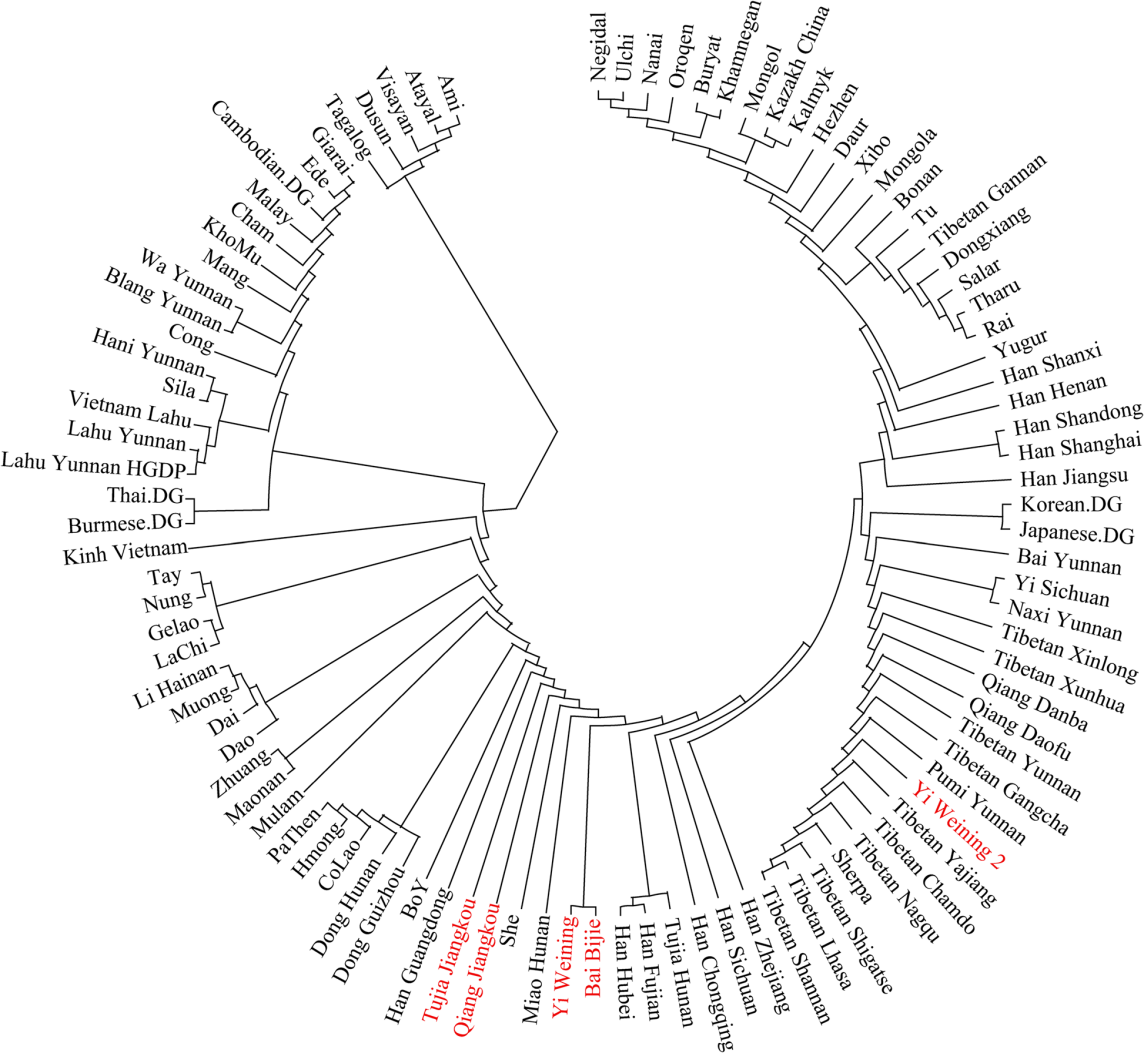
Phylogenetic (Neighbor-joining) tree based on Fst genetic distance between TBG groups (in red text) and reference populations in EA

We also computed the Fst genetic distance for our newly reported TBG and other populations in EA. In the phylogenetic tree (Fig. 5), our studied TBG populations clustered in the Sinitic and Tibeto-Burman branches with the southern populations, suggesting that they shared more genetic drift with the Sinitic and the Tibeto-Burman-speaking populations in the south. In addition, Bai and Yi groups clustered from the phylogenetic tree, while Qiang and Tujia groups clustered together. Bai and Yi groups were collected in west Guizhou, while Qiang and Tujia groups were collected in east Guizhou. There might be a genetic diversity in the TBG populations. The “Yi_Weining_2” group clustered with the Tibetan groups in the high-altitude region.

### Genetic continuity by the *f* statistics of populations

**Fig. 6:**
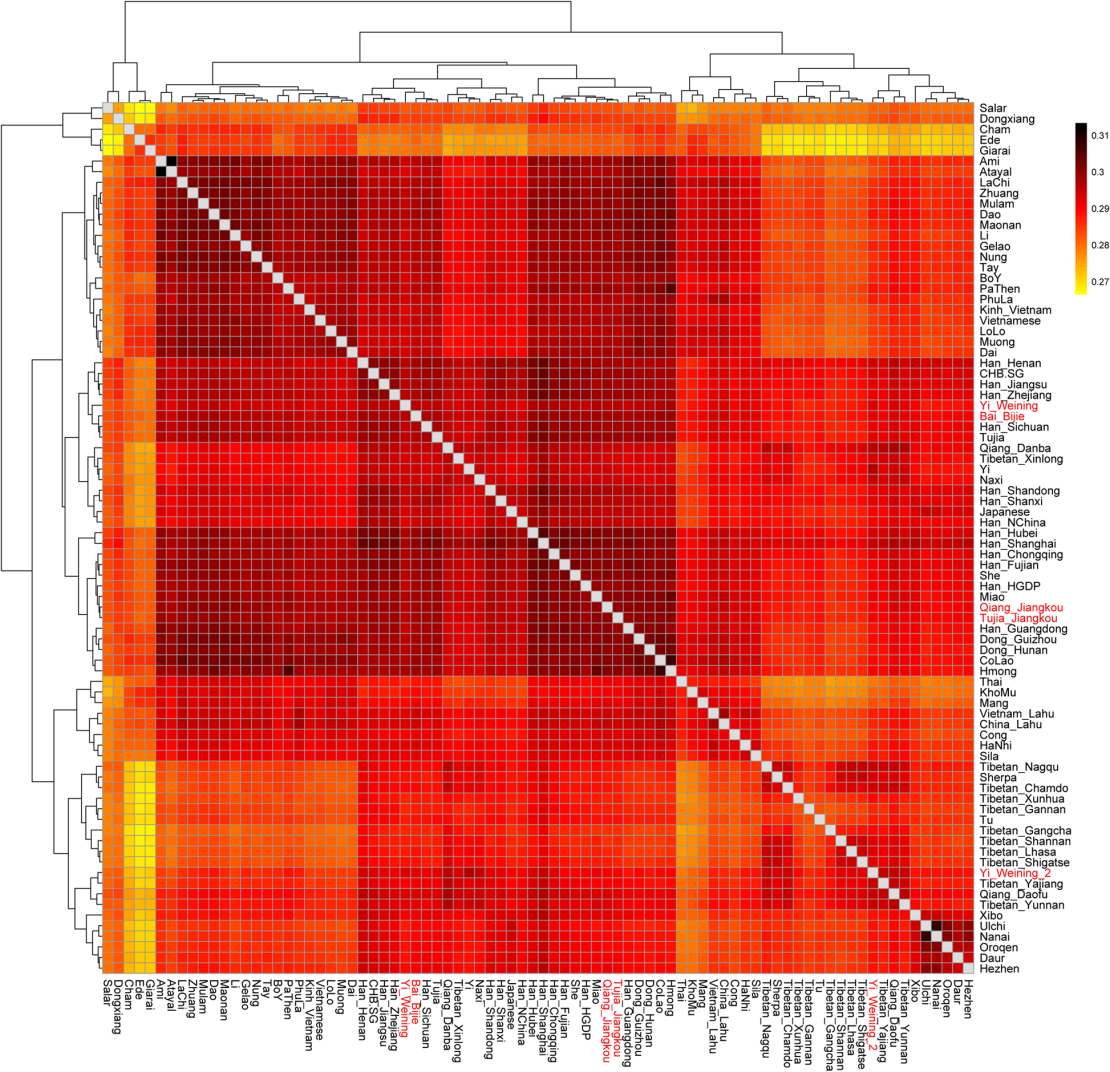
Heatmap of Outgroup-*f*_*3*_ statistics of the form *f*_*3*_ (X, Y; Mbuti) with Mbuti as Outgroup. TBG groups are in red text

Firstly, we calculated the Outgroup-*f*_*3*_ statistics in the form of *f*_*3*_ (X, Y; Mbuti) to quantify the population differentiation across EA discovered by PCA and ADMIXTURE. We showed the results of Outgroup-*f*_*3*_ statistics in Fig. 6. In the result, we found a southern populations cluster, a Tibeto-Burman and Han cluster, a Tibetan groups cluster, and an Altaic populations cluster. Our newly reported TBG populations, including Bai, Qiang, Yi, and Tujia, shared more genetic drifts with the Han groups. However, the “Yi_Weining_2” group clustered together with the high-altitude Tibetan populations. This was consistent with the PCA and ADMIXTURE. Moreover, the Bai and Yi groups clustered together, while the Qiang and Tujia groups clustered together. The results indicated a strong genetic affinity between the TBG and Han Chinese groups.

From the previous analysis, the TBG were genetically between the Tibetan and the southern groups. To explore the genetic relationships between the Tibetan groups and southern groups with the TBG, we carried out the *f*_*4*_ statistics in the form *f*_*4*_ (Tai-Kadai/Austronesian, Mbuti; Test, Tibetan) and *f*_*4*_ (Tibetan, Mbuti; Test, Tai-Kadai/Austronesian) (Supplementary Tables 1 and 2). We observed significant positive signals in both two *f*_*4*_ statistics. The positive *f*_*4*_ values demonstrated that compared to Tibetan, our studied TBG groups shared significantly more alleles with the southern populations related to Tai-Kadai and Austronesian and compared to Tai-Kadai or Austronesian-speaking populations in the south, our studied TBG groups shared significantly more alleles with the Tibetan populations. Our reported TBG populations were genetically between the high-altitude Tibetan and low-altitude southern groups in EA. As we know, the Tibetan groups contained a large proportion of genetic ancestry component from the Yellow River farming groups. Therefore, we next performed another two *f*_*4*_ statistics to test it. We found positive signals both in *f*_*4*_ (Yellow River farmers, Mbuti; Test, Tibetan) and *f*_*4*_ (Yellow River farmers, Mbuti; Test, Tai-Kadai/Austronesian), suggesting that TBG received genetic influence from the Yellow River farming groups in the north (Supplementary Tables 3 and 4).

### The southern and southeast coastal genetic influence in Tibeto-Burman speaking populations of Guizhou in Southwest China

**Fig. 7:**
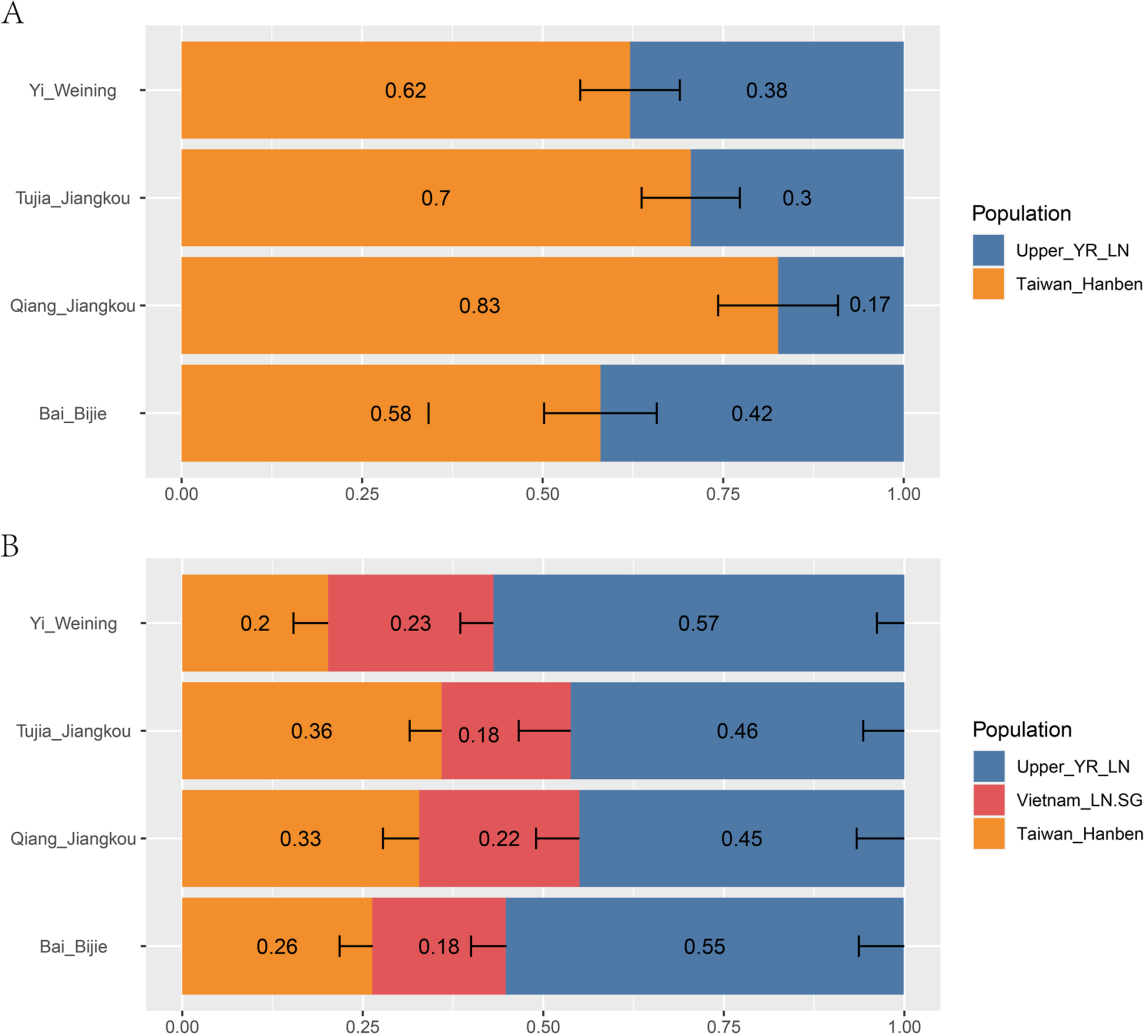
Graph results of *qpAdm* admixture models for TBG groups. **A** 2-way admixture models. **B** 3-way admixture models. Error bars denote standard error estimated using jackknife resampling

TBG were north–south admixtures. To identify the possible related source models for the TBG, we explored *qpAdm*-based admixture models. Firstly, we analyzed the ancestry component using the 2-way admixture models for the north–south interactions (Supplementary Table 5, Fig. 7A). We used the following outgroups: Mbuti, French, Anatolia_N, Onge, Yana_UP, Mongolia_N_East, Malaysia_LN, and Ust_Ishim. Our studied TBG populations in Southwest China were suggested to have driven 17%—42% of genetic ancestry from the Yellow River farming groups in the north. The left genetic components were from southern groups in EA.

Next, we continued exploring whether the southern component was from inland or coastal groups. We used Vietnam groups in the Late Neolithic as the inland source population. Therefore, we simulated the ancestral components by 3-way admixture models using the following outgroups: Mbuti, French, Papuan, Onge, DevilsCave_N, Japan_Jomon, Malaysia_LN, Atayal (Supplementary Table 5, Fig. 7B). We found that both inland and coastal ancestral groups have influenced TBG in the southern EA. Our reported TBG groups showed 45%—56.9% genetic ancestry from the Yellow River farming groups in the north. The TBG groups have driven 17.9%—22.9% genetic ancestry from mainland SEA and 20.2%—35.9% genetic ancestry from the southeast coastal EA. The genetic component from the Yellow River farming groups in the north drives the largest proportion of the TBG groups. The TBG were admixtures of groups from both the north and south, while the southern genetic influence was about half.

We also made some other *qpAdm* analyses for the southern genetic influence. We used other ancient human groups in MSEA and the southeast coastal EA. The results are presented in Supplementary Table 11. The outgroups set of these models were consistent with the 3-way *qpAdm* models in the above. Some of these models were fitted, while others were incorrect. The fitted models also provided that the southern genetic influence was both from the MSEA and the southeast coastal region of EA.

### Additional southeast coastal genetic influence on the Tibeto-Burman-speaking populations in Guizhou

**Fig. 8:**
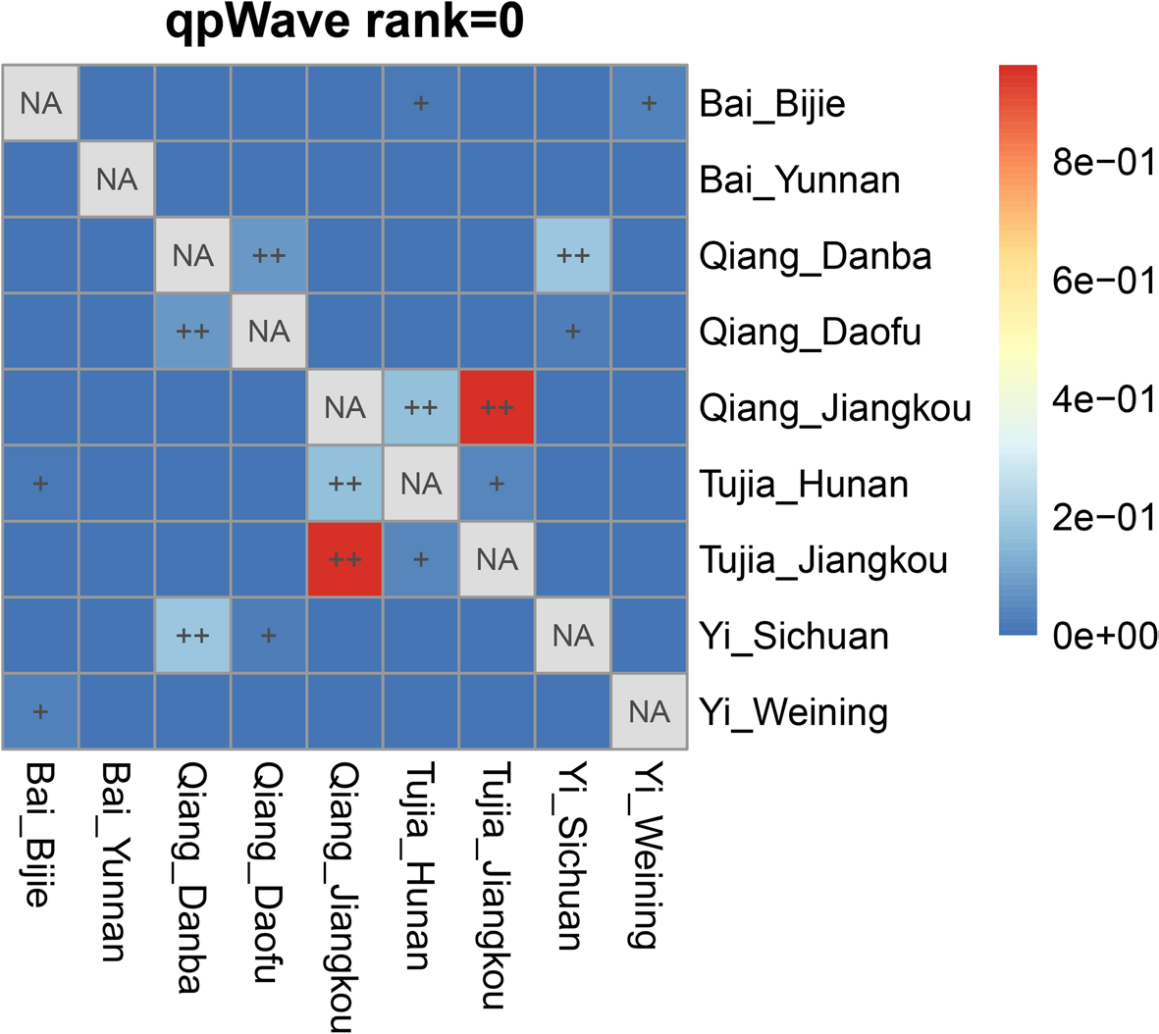
Heatmap of genetic homogeneity by pairwise *qpWave*. Heatmap shows *p*-values at rank = 0; *p*-value > 0.05 represented as “ +  + ”; *p*-value > 0.01 and < 0.05 represented as “ + ”

The TYC region is the key region for north-to-south interactions in EA, and many Tibeto-Burman-speaking populations live there. We carried out the *f*_*4*_ statistics of the form (Mbuti, Southeastern coastal; Guizhou Tibeto-Burman, Tibetan-Yi Corridor groups) to test whether the TBG and TYC groups had a different genetic affinity to the Southeast coastal groups in EA. The results (Supplementary Table 6) with significantly negative signals provided that compared to the TYC groups, the newly reported TBG shared more alleles with Southeast coastal groups. Next, we carried out the pairwise *qpWave* for our studied TBG groups and the Tibeto-Burman groups in the TYC to test whether they were genetically homogeneous. We used the following outgroups: Mbuti, French, Papuan, DevilsCave_N, Japan_Jomon, Atayal, and Onge. The results are presented in Fig. 8. We could observe that Bai_Bijie in Guizhou and Bai_Yunnan in the TYC were not genetically homogeneous. Moreover, the Qiang_Jiangkou in Guizhou and Qiang_Danba or Qiang_Daofu in Sichuan were also not genetically homogeneous. Similarly, the Yi_Weining in Guizhou and Yi_Sichuan (Yi group in Liangshan) were not genetically homogeneous, too. Bai_Yunnan (Dali), Qiang_Danba, Qiang_Daofu, and Yi_Sichuan were typical Tibeto-Burman-speaking populations in TYC. Our TBG populations were not genetically homogeneous with them. Therefore, there might be some additional gene flow for the reported TBG groups. We next aimed to explore the potential additional gene flow for the TBG groups.

**Fig. 9:**
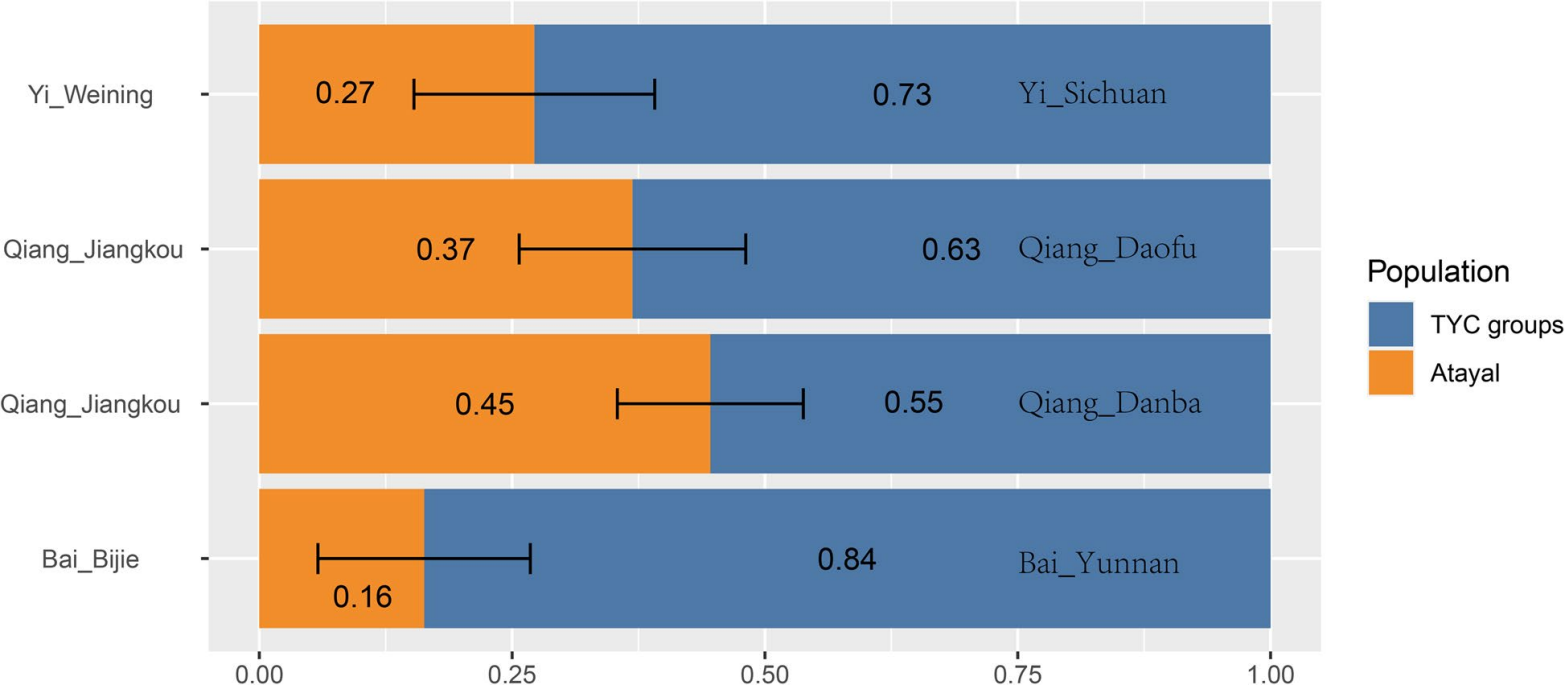
Graph results of *qpAdm* (2-way) admixture model for Tibeto-Burman groups in Guizhou and the TYC. Error bars denote standard error estimated using jackknife resampling

In Supplementary Table 6, we found that compared to the TYC groups, our studied TBG shared more alleles from southeast coastal groups. We used the *qpAdm* to model the admixtures. The TBG could be admixtures of the Tibeto-Burman populations in TYC and the Atayal-related genetic component from the southeast coastal region of EA. The admixture models were presented to show the results (Supplementary Table 7, Fig. 9). We observed from the *qpAdm*-based admixture models that the TBG populations contained 55.4%—83.7% genetic component from the TYC region and 16.3%—44.6% genetic component from the southeast coastal EA. The TBG had additional gene flow from the southeast coastal EA compared to the Tibeto-Burman-speaking groups in the TYC.

In addition, we compared the TBG with the Tibeto-Burman groups in Vietnam (Supplementary Table 9). From the *f*_*4*_ statistics, the LoLo group in Vietnam shared more alleles with populations from the southeast coastal region than Bai_Bijie. Bai_Bijie shared more alleles with populations from the southeast coastal region than the HaNhi, Vietnam_Lahu, Sila, and Cong groups in Vietnam. Qiang_Jiangkou in Guizhou shared more alleles with populations from the southeast coastal region than five Tibeto-Burman-speaking populations in Vietnam. The LoLo and PhuLa groups in Vietnam shared more alleles with groups from the southeast coastal region than Yi-Weining in Guizhou. While Yi_Weining shared more alleles with groups from the southeast coastal region than HaNhi and Sila groups in Vietnam. Tujia_Jiangkou in Guizhou shared more alleles with populations from the southeast coastal region than five Tibeto-Burman-speaking populations in Vietnam. The Tujia_Jiangkou and Qiang_Jiangkou were similar in the results. These results suggested that the Tibeto-Burman populations in Vietnam also had genetic diversity caused by the different proportions of the genetic ancestry from the southeast coastal groups in EA.

### The genetic diversity between the east and west Tibeto-Burman populations in Guizhou

Our studied TBG comprised four groups. Figure 1 shows that the Bai and Yi groups were collected in west Guizhou, while the Qiang and Tujia groups were collected in east Guizhou. Hence, the Bai and Yi called the west Guizhou Tibeto-Burman populations, while the Qiang and Tujia called the east Guizhou Tibeto-Burman populations. The phylogenetic tree indicated that the east and west Guizhou Tibeto-Burman groups were diverse genetically. Then, from the Outgroup-*f*_*3*_ results, the Bai and Yi groups were clustered together, while the Qiang and Tujia groups were clustered together. Next, we conducted *f*_*4*_ statistics to test the different genetic affinity between the east and west Guizhou Tibeto-Burman populations. The results (Supplementary Table 8) with significantly negative signals in *f*_*4*_ (Southeast coastal, Mbuti; West Guizhou Tibeto-Burman, East Guizhou Tibeto-Burman) showed that the east Guizhou Tibeto-Burman populations had a stronger genetic affinity to the southeast coastal populations than the west Guizhou Tibeto-Burman groups. As a result, it suggested a genetic difference between the TBG mainly caused by the different proportions of the genetic components from the southeast coastal region of China. It indicated that the expansion of the genetic influence of the southeast coastal groups had a genetic gradient in Southwest China.

## Discussion

Since the Neolithic, multiple waves of population migrations and cultural interactions have shaped the genetic structure in the southwest region of China [6]. The Tibeto-Burman ancestries originated from the northwest region of China. A part of them spread southward, mainly via the TYC. The TYC is a meeting point between many EA populations, especially those closely related to the Han, Tibetan, and southern EAs. The Yellow River farming groups primarily influenced the northern TYC. In contrast, the southern TYC was influenced strongly by the southern EA groups with the expansion of rice-farming agriculture since the Middle Neolithic. The rice-farming expansion had deeply influenced the genetic profile of the southern Tibeto-Burman populations, suggesting migrations from east to west through both inland and coastal routes. However, many previous studies focused on the TYC. Guizhou region needed more dense sampling and fine-scale genetic analysis.

In this study, we generated and analyzed genome-wide SNP data from modern human groups encompassing 4 TBG. We comprehensively compared and analyzed our newly reported TBG populations with the previously published ancient and modern worldwide populations to infer Guizhou's detailed genetic profile and demographic admixture history.

We found the genetic affinity of TBG with the surrounding Tibetans, southern Tai-Kadai and Austronesian groups, and northern farming groups in the Yellow River Basin in the Neolithic Age from the PCA pattern. The Yi group was closer to the Tibetans in the high-altitude region. Other ethnic groups in our research were related closely to Han groups. The results of the ADMIXTURE analysis also proved it. From the result, the Yi group contained more Tibetan-related ancestral components than others, suggesting its closer genetic history with the TYC region. From the *f*_*4*_ statistics, the TBG received genetic contributions from many human groups, including the northern farmer and hunter-gatherers and southern EA and SEA mainland populations. The TBG were like the Tibeto-Burman speakers in Yunnan. Moreover, we explored the gene flow from proper ancestral populations of our reported groups and reconstructed these populations' deep genetic history in the Guizhou region.

Our study revealed that demographic migrations and cultural interactions from north to south and east to west since the Late Neolithic had shaped the genetic structure of populations in the Guizhou region—this region’s dominant genomic legacy associated with the millet-farming groups in the Yellow River in northern China. The TBG were influenced by the expansion of rice-farming groups from both the mainland SEA (Vietnam_LN) and the southeast coastal EA region (Taiwan_Hanben). The *qpAdm*-base admixture models (Fig. 7) provided evidence of it. Figure 7A indicated that the Qiang group in Guizhou contained more southern genetic components than other groups, showing their extended admixture history with the southern populations in EA. Yi group has more northern genetic components, revealing their relatively conservative lifestyle in the past years because they received less southern genetic ancestry component. Bai group also had more northern genetic ancestry from the *qpAdm*. The results were consistent with the geographical area because Bijie was located west of Guizhou and closer to the TYC region.

Bai, Qiang, and Yi groups were the typical Tibeto-Burman-speaking populations in the TYC region. However, the Bai, Qiang, and Yi groups in Guizhou were not genetically homogeneous with them. The vast mountains and rivers between Guizhou and the TYC limited the population interactions. Our results indicated that compared to the Tibeto-Burman populations in the TYC, TBG composed additional gene flow from the southeast coastal EA populations. Due to complex geographical factors, the westward expansion of the rice-farming groups of the southeast coastal region was decelerated in the southwest region of China. The precipitous mountain ranges and rivers were the natural barriers to human gene flow.

And there was a genetic diversity in TBG. Bai and Yi were the west Tibeto-Burman groups in Guizhou, while the Qiang and Tujia were the east part. This can be observed in the sampling map in Fig. 1. In the ADMIXTURE result, the west and east parts of them presented different genetic structures. In the phylogenetic tree and outgroup-*f*_*3*_ results, the Bai and Yi groups were clustered together, while Qiang and Tujia groups were clustered together. The pairwise *qpWave* testing (Fig. 8) showed that the Qiang_Jiangkou and Tujia_Jiangkou were genetically homogeneous. However, the Bai_Bijie and Yi_Weining were not genetically homogeneous. There might be some additional gene flow to one of them. Supplementary Table 8 showed that the east part of Tibeto-Burman populations shared more genetic components from the southeast coastal region than the west part. This result supported the genetic diversity in the TBG caused mainly by the different proportions of the genetic components from southeast coastal China. It suggested that the vast mountains and rivers in Southwest China might decelerate the westward expansion of the Southeast coastal rice-farming groups. Previous studies have shown a genetic substructure in Tai-Kadai populations in Guizhou mainly caused by the different proportions of the genetic components from the Yellow River millet-farming groups in the north [50]. In our study, the genetic differences in TBG were caused mainly by the different proportions of the genetic influences from the south. It suggested a genetic gradient of the expansion of the southeast coastal populations in West China. It provided that the Guizhou region was a complex area influenced by different populations from different regions with different genetic component proportions.

Our results were consistent with the archaeological and ethnolinguistic evidence, supporting the Neolithic human migration and cultural intercourse from northern China to the southwest region. The northern genetic component of our studied TBG populations showed a connection with the Neolithic culture from Yellow River millet-farming groups, supporting the viewpoint of the Northern China origin of Sino-Tibetan speaking populations inferring from language genealogy [14]. Some proto-Tibeto-Burman populations expanded southward to the southwest through the TYC pathway [51]. Some of them then migrated eastward into the Guizhou region. The populations in Guizhou had admixed with the southward expansion of millet-farming groups and the westward and northward expansion of rice-farming groups in southern EA. They appeared to have a similar genetic structure to the Tibeto-Burman speakers in Yunnan. However, compared to Yunnan groups, TBG shared more genetic components from the southeast coastal groups of China. Our results supported the east–west interactions in southern China and the interaction between EA and SEA. Recent genetic studies also supported the coastal connection route from Southeast China to MSEA, including present-day Vietnam [22, 52]. However, the coastal connection route was controversial until the present. The southeast coastal groups also influenced the Tibeto-Burman populations in Vietnam to a different degree. From the PCA plot, the ancient groups in Guangxi in the Neolithic were close to the ancient groups in MSEA. Our results also suggested a northward migration from the rice-farming groups from the Mainland SEA into Southwest China. However, the genetic influence of southeast coastal China played a significant role in the formation of different genetic structures in Southwest China. Multiple waves of migrations from northern China, the southern EA coastal region, and the SEA mainland reshaped the genetic profile of the TBG in Southwest China. Our results primarily provided the different proportions of the genetic influence from the southeast coastal groups in EA. The westward expansion of the southeast coastal groups also needs some ancient genomic evidence to support it.

We note that the limitations of the overlapped SNPs between the different datasets might hinder the dissection of the population admixture history. More investigations and high-depth whole-genome sequencing data could provide more precise information about the genetic profile and demographic admixture history in West China.

### Supplementary Information

**Additional file 1: The supplementary tables: Supplementary table 1.** *f*_*4*_ (Tai-Kadai/Austronesian, Outgroup; Test, Tibetan). **Supplementary table 2.** *f*_*4*_ (Tibetan, Outgroup; Test, Tai-Kadai/Austronesian). **Supplementary table 3.** *f*_*4*_ (Yellow River groups, Outgroup; Test, Tibetan). **Supplementary table 4.** *f*_*4*_ (Yellow River groups, Outgroup; Test, Tai-Kadai/Austronesian). **Supplementary table 5.** Two-way and three-way qpAdm models of studied Tibeto Burman populations. **Supplementary table 6.** *f*_*4*_ (Outgroup, Southeast coastal; Guizhou Tibeto-Burman, Tibetan-Yi Corridor groups). **Supplementary table 7.** Admixture models of Tibeto-Burman groups for southeast coastal gene flow. **Supplementary table 8.** *f*_*4*_ (Southeast coastal, Outgroup; West Guizhou Tibeto-Burman, East Guizhou Tibeto-Burman). **Supplementary table 9.** *f*_*4*_ (Outgroup, Southeast coastal; Guizhou Tibeto-Burman, Vietnam Tibeto-Burman). **Supplementary table 10.** Information for the reference populations. **Supplementary table 11.** Other three-way qpAdm models with different ancient groups in MSEA and the southeast coastal EA.

## Data Availability

The datasets presented in this study can be found in online repositories. The names of the repository/repositories and accession number(s) can be found below: https://zenodo.org/record/7730960.
